# Spatially Controlled Highly Branched Vinylsilicones

**DOI:** 10.3390/polym13060859

**Published:** 2021-03-11

**Authors:** Mengchen Liao, Yang Chen, Michael A. Brook

**Affiliations:** Department of Chemistry and Chemical Biology, McMaster University, 1280 Main St. W., Hamilton, ON L8S 4M1, Canada; liaom6@mcmaster.ca (M.L.); dychen@mcmaster.ca (Y.C.)

**Keywords:** silicone polymers, dendritic branches, Piers–Rubinsztajn reaction, hydrosilylation

## Abstract

Branched silicones possess interesting properties as oils, including their viscoelastic behavior, or as precursors to controlled networks. However, highly branched silicone polymers are difficult to form reliably using a “grafting to” strategy because functional groups may be bunched together preventing complete conversion for steric reasons. We report the synthesis of vinyl-functional highly branched silicone polymers based, at their core, on the ability to spatially locate functional vinyl groups along a silicone backbone at the desired frequency. Macromonomers were created and then polymerized using the Piers–Rubinsztajn reaction with dialkoxyvinylsilanes and telechelic HSi-silicones; molecular weights of the polymerized macromonomers were controlled by the ratio of the two reagents. The vinyl groups were subjected to iterative (two steps, one pot) hydrosilylation with alkoxysilane and Piers–Rubinsztajn reactions, leading to high molecular weight, highly branched silicones after one or two iterations. The vinyl-functional products can optionally be converted to phenyl/methyl-modified branched oils or elastomers.

## 1. Introduction

Macromolecules comprised of dendritic structures have been widely utilized in a variety of different areas both in academia and industry [1,2,3]. The introduction of, normally, randomly dispersed dendritic units with multi-functionalities along the polymeric backbone is key to the unique physiochemical properties they possess [4,5]. In comparison with their linear counterparts, highly branched materials exhibit low viscosity and enhanced solubility, and the higher density branches can optionally possess functional groups to allow further modification [6]. The relative simplicity of the synthetic routes to dendritic branches differentiates them from the perfectly structured dendrimers that require laborious synthesis and work-up procedures; the higher the generation number, the more likely defects are to be generated. The former approach is, therefore, more suitable for industrial scale-up [7]. In light of their unique properties, highly branched and hyperbranched polymers have been commercialized for applications ranging from coatings, additives, and insulators to biomedical applications [8,9,10,11,12].

Dendritic moieties are, by definition, sterically demanding. As a consequence, when forming dendritic branches using a “grafting to” process on a polymer backbone, it can be difficult to achieve a complete reaction because: (i) the backbone provides steric encumbrance that decreases the likelihood of successful grafting, a situation that, (ii) is made worse when functional groups on the backbone are found in close proximity. The random dispersion of the dendritic units throughout the macromolecules leads to the formation of irregular structures [13]. Would a synthetic benefit be realized by controlling spatial location of the dendritic branches, and would spatial distribution contribute to useful properties?

**Figure 1:**
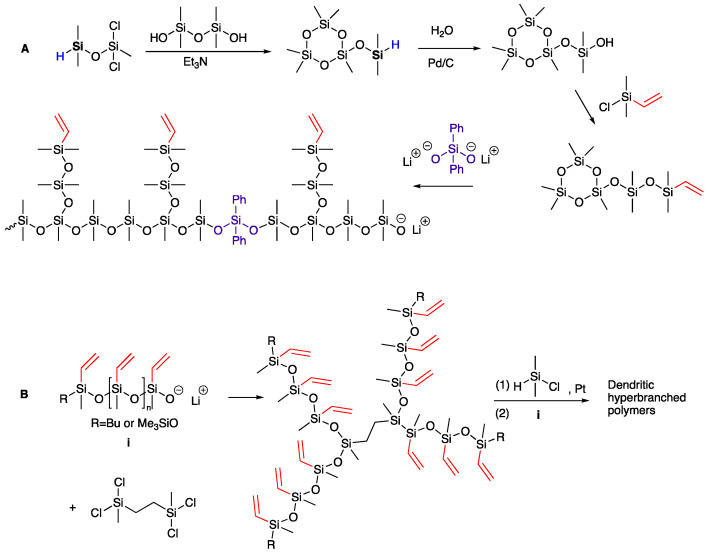
(**A**) The synthesis of hyperbranched polysiloxane using anionic ring-opening polymerizations [19]; (**B**) The synthesis of dendrite-branched polysiloxanes [21].

Polysiloxanes are renowned for their flexibility, biocompatibility, gas permeability, low T_g_, hydrophobicity, and low surface energy [14,15,16]. The initial investigations of hyperbranched polysiloxanes were conducted by Muzafarov and co-workers through condensation of triethoxysilanol, catalyzed by ammonia, that yielded a transparent polymer [17,18]. In 2000, Paulasaari and Weber established lithium silanolate-initiated anionic polymerization (Figure 1A) for the preparation of a vinyl-containing hyperbranched polysiloxane with a molecular weight (MW) of 29,050 g mol^−1^ (vinyl = Vi = ~HC = CH_2_) [19,20]. Chojnowski and co-workers further advanced this methodology with the synthesis starting from vinyl-substituted cyclotrisiloxanes to give vinyl-containing branched polysiloxanes of various topologies, including star- to dendritic-branched architectures (Figure 1B) [21].

**Figure 2:**
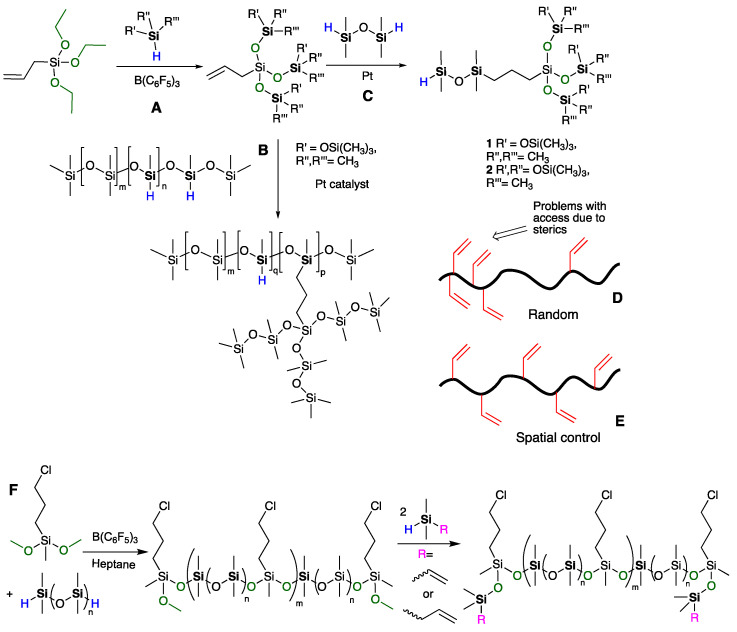
The preparation of (**A**,**C**): well-defined silicone dendrons via an iterative process [24]. (**B**) Higher branched polymers from random SiH precursors. Model comparison of vinylsilicones with either (**D**) random or (**E**) controlled locations. (**F**) Using the Piers–Rubinsztajn reaction to prepare spatially controlled chloropropylsilicones [26].

The Piers–Rubinsztajn reaction (PR), which provides a facile route to structurally complex silicones, involves the reaction of hydrosilanes with the strong Lewis acid catalyst B(C_6_F_5_)_3_ in the presence of appropriate nucleophiles [22]. We previously reported the use of this process combined with hydrosilylation reactions to synthesize precise silicone dendrimers with molar mass of up to 13,500 g mol^−1^ using iterative reactions of PR and platinum-catalyzed hydrosilylation in the absence of degradation (Figure 2A,C) [23]. Well-defined silicone resins with tunable branching degrees could also be prepared using this pair of reactions [24].

We wished to create highly branched linear silicone polymers with different branching densities to take advantage of the benefits these polymers convey. While synthesis of the monomer branches was straightforward (Figure 2A,C), it was not always possible to achieve complete grafting of highly branched species onto randomly located SiH functional groups typically found on commercial silicones (unpublished data, Figure 2B); this inefficiency was ascribed to the steric hinderance in zones of high functional group density on the backbone (Figure 2D). Such inhomogeneity is unavoidable in functional silicones prepared by traditional redistribution reactions [25]. We reasoned two factors could be used to decrease sensitivity to steric issues: (i) move the locus of reaction further away from the silicone backbone by use of vinyl groups on the backbone instead of SiH groups, and (ii) control the spatial distribution of vinyl groups to ensure adjacent functional groups are avoided (Figure 2E). In the dendrimeric vernacular, this refers to divergent rather than convergent growth or “grafting from” rather than “grafting to”.

The concept of properly controlling polymer synthesis using the perspective of synthetic organic chemists is gaining traction [27]. In the case of silicones, Skov and co-workers initially reported a strategy for synthesizing siloxane copolymers with spatially distributed pendent functional groups, including alkyl chloride and alkyl azide, using the PR reaction [26]. Such polymers should be less sensitive to steric issues during further functionalization because the sequence ensures functional groups maintain a constant distance along the backbone (Figure 2E,F). Using this strategy, we describe preparation of a hierarchical library of precisely spaced vinyl, or alkoxy, pendent linear silicones with either SiH or methoxy at the termini via iterative PR condensation and platinum-catalyzed hydrosilylation. The products include polymers bearing both SiH and SiVinyl groups, which are difficult to prepare by traditional routes. The polymers underwent self-crosslinking in a one-pot process to give soft elastomers possessing residual vinyl groups that, we show, can undergo secondary modification with different silicones. We expanded this concept to create a library of silicone crosslinkers possessing dendritic branches with different degrees of vinyl and alkoxy functionalization and show that, at lower generations, steric problems with functionalization are avoided.

## 2. Materials and Methods

### 2.1. Materials

Pentamethyldisiloxane (Me_3_SiOSiMe_2_H, MM^H^), bis(trimethylsiloxy)methylsilane ((Me_3_SiO)_2_SiMeH, MD^H^M), tetramethyldisiloxane (**H-0-H** HMe_2_SiOSiMe_2_H, M^H^M^H^), phenyldimethylsilane, hydride-terminated phenyl pendent PDMS (PMS-H03, 370 g mol^−1^), and vinyltetramethyldisiloxane were purchased from Gelest and used as received. Hydride-terminated PDMS (**H-13-H** DMS-H03, MW = 1100 g mol^−1^; **H-18-H** DMS-H11, MW = 1500 g mol^−1^; **H-77-H** DMS-H21, MW = 5800 g mol^−1^; **H-285-H** DMS-H25, MW = 21,200 g mol^−1^; DMS-H31, MW = 24,400 g mol^−1^) were purchased from Gelest and were vacuum-distilled (kugelrohr) at 120 °C for 1 h prior to use. Dimethoxymethylvinylsilane (97%), triethoxysilane, and platinum(0)-1,3-divinyl-1,1,3,3-tetramethyldisiloxane complex solution (Karstedt’s catalyst) in xylene (Pt 2%) were obtained from Sigma Aldrich and used as received. B(C_6_F_5_)_3_ was purchased from Alfa Aesar. Toluene (solvent) received from Caledon (HPLC grade) was dried over activated alumina before use. Deuterated NMR solvent (CDCl_3_) was obtained from Cambridge Isotope Laboratories. The glass apparatus was dried overnight at 120 °C and cooled in a dry nitrogen atmosphere for 30 min prior to use.

### 2.2. Methods

^1^H NMR spectra were recorded on a Bruker AV600 MHz spectrometer using deuterated solvents (CDCl_3_). For gel permeation chromatography (GPC), a Viscotek GPC Max (VE 2001 GPC Solvent/Sample Module) was used to determine molecular weights. The system was equipped with a Viscotek VE 3580 RI Detector, a Viscotek 270 Dual Detector, and a PolyAnalytik SupeRes PAS-101 (8 mm × 30 cm) column with a single pore, styrene-divinylbenzene gel, 6 μm particle size. Toluene was used as the eluent at a flow rate of 1.0 mL min^−1^. Refractive index (R_f_) measurements were carried out with a VEE GEE Scientific Abbe Refractometer Model C10.

### 2.3. Synthesis

#### 2.3.1. General Procedures for the Preparation of Vinylsilicones with Controlled Spacing, Shown for P-Vi-14

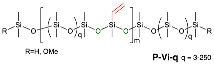



Hydride-terminated PDMS (**H-13-H**, 43.56 g, 39.60 mmol) was weighed into a 1.0 L three-neck round-bottomed flask with dry hexanes (218.0 mL). The flask was flushed with nitrogen before capping with a septum and left under a positive pressure of dry nitrogen. B(C_6_F_5_)_3_ stock solution (0.079 mL, 51.2 mg mL^−1^ in dry toluene, 0.0079 mmol) was added before adding excess dimethoxymethylvinylsilane (6.29 g, 47.57 mmol). The mixture was stirred for 3h at room temperature before neutral alumina was added to the flask to quench the B(C_6_F_5_)_3_ catalyst, followed by filtration through Celite and rinsing with hexanes. **P-Vi-14** was obtained by removing hexanes under reduced pressure (rotavap at 60 °C) with yield of 83.8% (41.77 g) and vinyl conc. of 7% calculated based on the ^1^H NMR data (sample calculation, see Supporting Information, SI).

^1^H NMR (600 MHz, chloroform-*d*): δ 5.77–6.02 (m, 3H, SiC*H* = C*H*_2_), 3.47–3.48 (s, 0.10H, SiOC*H*_3_), 0.05–0.13 (m, 83.55H, SiC*H*_3_) ppm; GPC: M_n_ = 37,400 g mol^−1^; M_w_ = 69,300 g mol^−1^; *Đ_M_* = 1.85.

#### 2.3.2. Capping OMe Groups in P-Vi-14

**P-Vi-14** (10.63 g, 0.28 mmol) and pentamethyldisiloxane (MM^H^, 0.169 g, 1.15 mmol) were added to a pre-dried 25 0.0 mL round-bottomed flask together with dry hexanes (50.0 mL). The reaction flask was capped and flushed with dry nitrogen. B(C_6_F_5_)_3_ stock solution (0.114 mL, 5.12 mg mL^−1^ in dry toluene, 0.00114 mmol) was added. The mixture was stirred for 3h at room temperature before neutral alumina was added to the flask to quench the B(C_6_F_5_)_3_ catalyst, followed by filtration through Celite and rinsing with hexanes. The capped product was obtained by removing hexanes under reduced pressure with yield of 97.6% (10.54 g) and vinyl conc. of 7% based on ^1^H NMR data.

^1^H NMR (600 MHz, chloroform-*d*): δ 5.77–6.04 (m, 3H, SiC*H* = C*H*_2_), 0.05–0.19 (m, 84.62H, SiC*H*_3_) ppm; GPC: M_n_ = 39,000 g mol^−1^; M_w_ = 73,700 g mol^−1^; *Đ_M_* = 1.89.

#### 2.3.3. Synthesis of Compound P-Vi-14-OR_3_

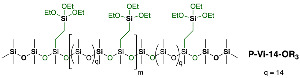



The capped compound from the previous step (10.38 g, 0.27 mmol) and triethoxysilane (4.88 g, 29.71 mmol) were added to a pre-dried 250.0 mL round-bottomed flask together with dry hexanes (30.0 mL). The reaction flask was capped and flushed with dry nitrogen. Karstedt’s catalyst (0.015 mL, 2 wt% in xylene) was then directly added from the bottle. The mixture was stirred at room temperature for 24 h at room temperature before activated carbon was added to the flask to quench the Karstedt catalyst followed by filtration through Celite and rinsing with hexanes. **P-Vi-14-OR_3_** was obtained by removing hexanes under reduced pressure with yield of 75% (11.43 g).

^1^H NMR (600 MHz, chloroform-*d*): δ 3.79–3.83 (m, 6H, SiOC*H*_2_CH_3_), 1.20–1.23 (m, 9.04H, SiOCH_2_C*H*_3_), 0.50–0.59 (m, 3.43H, SiC*H*_2_-C*H*_2_-Si), 0.06–0.08 (m, 78.53H, SiC*H*_3_) ppm; GPC: M_n_ = 99,300 g mol^−1^; M_w_ = 185,400 g mol^−1^; *Đ_M_* = 1.87.

#### 2.3.4. Synthesis of Compound P-Vi-14-OSi_3_

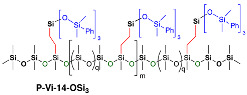



**P-Vi-14-OR_3_** (0.088 g, 0.0053 mmol) and phenyldimethylsilane (0.07 g, 0.514 mmol) were added to a pre-dried 25.0 mL round-bottomed flask together with dry hexanes (5.0 mL). The reaction flask was capped and flushed with dry nitrogen. B(C_6_F_5_)_3_ stock solution (0.0197 mL, 5.12 mg mL^−1^ in dry toluene, 0.000197 mmol) was added. The mixture was stirred for 3 h at room temperature before neutral alumina was added to the flask to quench the B(C_6_F_5_)_3_ catalyst followed by filtration through Celite and rinsing with hexanes. **P-Vi-14-OSi_3_** was obtained by removing hexanes under reduced pressure with yield of 66% (0.11 g).

^1^H NMR (600 MHz, chloroform-*d*): δ 7.32–7.54 (m, 24.57H, aromatic H), 0.41–0.42 (m, 4H, SiC*H*_2_-C*H*_2_-Si), 0.02–0.08 (m, 118.36H, SiC*H*_3_) ppm; GPC: M_n_ = 55,000 g mol^−1^; M_w_ = 116,500 g mol^−1^; *Đ_M_* = 2.12; R_I_ = 1.383.

#### 2.3.5. Synthesis of Compound P-Vi-14-OVi_3_

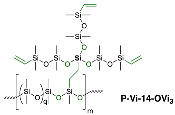



**P-Vi-14-OR_3_** (0.89 g, 0.053 mmol) and vinyltetramethyldisiloxane (1.38 g, 8.61 mmol) were added to a pre-dried 250.0 mL round-bottomed flask together in dry hexanes (25.0 mL). The reaction flask was capped and flushed with dry nitrogen. B(C_6_F_5_)_3_ stock solution (0.127 mL, 5.12 mg mL^−1^ in dry toluene, 0.00127 mmol) was added. The mixture was stirred for 3 h at room temperature before neutral alumina was added to the flask to quench the B(C_6_F_5_)_3_ catalyst followed by filtration through Celite and rinsing with hexanes. **P-Vi-14-OVi_3_** was obtained by removing hexanes under reduced pressure with yield of 76% (1.73 g).

^1^H NMR (600 MHz, chloroform-*d*): δ 5.71–6.16 (m, 9H, SiC*H* = C*H*_2_), 0.45–0.46 (m, 2.24H, SiC*H*_2_-C*H*_2_-Si), 0.06–0.16 (m, 93.34H, SiC*H*_3_) ppm; GPC: M_n_ = 278,800 g mol^−1^; M_w_ = 582,500 g mol^−1^; *Đ_M_* = 2.09. We attribute this higher than expected increase in MW to adventitious hydrolysis/condensation of the polymers.

#### 2.3.6. Synthesis of Compound P-Vi-14-OSi_3_OSi_9_

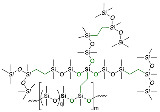



**P-Vi-14-OVi_3_** (0.33 g, 0.0012 mmol) and bis(trimethylsiloxy)methylsilane (MD^H^M, 0.37 g, 1.67 mmol) were added to a pre-dried 50.0 mL round-bottomed flask together with dry hexanes (25.0 mL). The reaction flask was capped and flushed with dry nitrogen. Karstedt’s catalyst (0.0075 mL, 2 wt% in xylene) was then directly added. The mixture was stirred for 24 h at room temperature before activated carbon was added to the flask to quench the Karstedt catalyst followed by filtration through Celite and rinsing with hexanes. **P-Vi-14-OSi_3_OSi_9_** was obtained by removing hexanes under reduced pressure with yield of 70% (0.49 g).

^1^H NMR (600 MHz, chloroform-*d*): δ 0.32–0.56 (m, 16H, SiC*H*_2_-C*H*_2_-Si), 0.01–0.09 (m, 178.59H, SiC*H*_3_) ppm; GPC: M_n_ = 310,500 mol^−1^; M_w_ = 609,200 g mol^−1^; *Đ_M_* = 1.96.

#### 2.3.7. Attempted Synthesis of Compound P-Vi-14-OVi_3_OR_9_, and then Branching

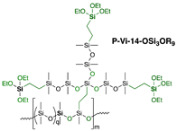



**P-Vi-14-OVi_3_** (0.079 g, 0.000283 mmol) and triethoxysilane (0.083 g, 0.51 mmol) were added to a pre-dried 50.0 mL round-bottomed flask together with dry hexanes (10.0 mL). The reaction flask was capped and flushed with dry nitrogen. Karstedt’s catalyst (0.0075 mL, 2 wt% in xylene) was then added. The mixture was stirred for 24 h at room temperature before activated carbon was added to the flask to quench the Karstedt catalyst followed by filtration through Celite and rinsing with hexanes to give, after removing hexanes under reduced pressure, compound **6** in a yield of 89% (0.14 g).

^1^H NMR (600 MHz, chloroform-*d*): δ 3.81–3.83 (m, 18H, SiOC*H*_2_CH_3_), 1.21–1.25 (m, 27.28H, SiOCH_2_C*H*_3_), 1.53 (s, 10.40H), 0.45–0.67 (m, 14.89H, SiC*H*_2_-C*H*_2_-Si), 0.04–0.08 (m, 113.63H, SiC*H*_3_) ppm; GPC: M_n_ = 56,400 g mol^−1^; M_w_ = 109,200 g mol^−1^; *Đ_M_* = 1.94.

#### 2.3.8. Attempted Branching of Compound P-Vi-14-OVi_3_OR_9_

**P-Vi-14-OVi_3_OR_9_** (0.159 g, 0.0028 mmol) and bis(trimethylsiloxy)methylsilane (MD^H^M, 1.779 g, 7.995 mmol) were added to a pre-dried 50.0 mL round-bottomed flask together with dry hexanes (20.0 mL). The reaction flask was capped and flushed with dry nitrogen. B(C_6_F_5_)_3_ stock solution (0.131 mL, 5.12 mg mL^−1^ in dry toluene, 0.00131 mmol) was added. The mixture was stirred for 24 h at room temperature before neutral alumina was added to the flask to quench the B(C_6_F_5_)_3_ catalyst. By ^1^H NMR, the conversion of the ethoxy groups is < 20%.

^1^H NMR (600 MHz, chloroform-*d*): δ 4.62–4.63 (m, 9.63H, Si*H*), 3.72–3.75 (m, 16.19H, SiOC*H*_2_CH_3_), 1.53 (s, 15.70H), 1.19–1.23 (m, 24.24H, SiOCH_2_C*H*_3_), 0.55–0.64 (m, 16H, SiC*H*_2_-C*H*_2_-Si), 0.02–0.12 (m, 2757.83H, SiC*H*_3_) ppm.

The mixture from the above procedure was further heated at 50 °C for 24 h and based on the ^1^H NMR, the maximum conversion of the ethoxy groups was 53%.

^1^H NMR (600 MHz, chloroform-*d*): δ 4.62 (m, 0.61H, Si*H*), 3.72–3.81 (m, 9.43H, SiOC*H*_2_CH_3_), 1.19–1.23 (m, 15.21H, SiOCH_2_C*H*_3_), 0.54–0.56 (m, 16H, SiC*H*_2_-C*H*_2_-Si), 0.02–0.10 (m, 2090.55H, SiC*H*_3_) ppm; GPC: M_n_= 18,000 g mol^−1^; M_w_ = 49,300 g mol^−1^; *Đ_M_* = 2.75. This low MW indicates that most of the material has been converted to a macroscopic resin, and only low MW materials remain in the solution.

#### 2.3.9. Synthesis of Silicone Elastomers Using Controlled Spacing Hydride-Terminated Vinyl-Containing Linear Silicone

**Step 1**: Synthesis of hydride-terminated, vinyl-pendent linear silicones (**P-Vi-28**)

Hydride-terminated PDMS (**H-18-H**, 1500 g mol^−1^, 1.03 g, 0.94 mmol) was weighed into a 250.0 L three-neck round-bottomed flask with dry hexanes (5.0 mL). The flask was flushed with nitrogen before being capped with a septum and left under positive nitrogen pressure. B(C_6_F_5_)_3_ stock solution (0.133 mL, 51.2 mg mL^−1^ in dry toluene, 0.0133 mmol) was added before adding dimethoxymethylvinylsilane (0.074 g, 0.560 mmol). The mixture was stirred for 3 h at room temperature before neutral alumina was added to the flask to quench the B(C_6_F_5_)_3_ catalyst followed by filtration through Celite and rinsing with hexanes. The product **P-Vi-28** was obtained by removing hexanes under reduced pressure with yield of 84% (0.93 g).

^1^H NMR (600 MHz, chloroform-*d*): δ 5.78–6.03 (m, 3H, SiC*H* = C*H*_2_), 4.70–4.74 (Si*H*, m, 0.11H), 0.07–0.45 (m, 164.66H) ppm; GPC: M_n_ = 65,700 g mol^−1^; M_w_ = 91,300 g mol^−1^; *Đ_M_* = 1.39.

**Step 2**: Self-cured silicone elastomer using **P-Vi-28** (**entry 3** Table 2)

The product obtained from **Step 1** (0.928 g, 0.014 mmol) and Karstedt’s catalyst (0.260 mL, 5 mg mL^−1^ in dry toluene) were added into a 24-well plate. After stirring, the mixture was then placed under vacuum at 80 °C for 3h.

**Step 3**: Synthesis of silicone elastomer 2:1 SiH/SiVinyl from **P-Vi-28** (**entry 5** Table 2)

The as-prepared vinyl containing elastomer (0.078 g, 0.042 mmol for Si^Vi^) was allowed to swell in toluene (2 mL) containing PMS-H03 (0.0158 g, 0.085 mmol for Si^H^) for 24 h in a 25 mL glass vial prior to the addition of Karstedt’s catalyst (0.0188 mL, 5 mg mL^−1^ in dry toluene).

OR **Step 3**: Synthesis of silicone elastomer 1:1 SiH/SiVinyl from **P-Vi-28** (**entry 6** Table 2)

The as-prepared vinyl containing elastomer (0.07 g, 0.038 mmol for Si^Vi^) was swollen in toluene (2 mL) and PMS-H03 (0.007 g, 0.038 mmol for Si^H^) for 24h in a 25 mL glass vial prior to the addition of Karstedt’s catalyst (0.0154 mL, 5 mg mL^−1^ in dry toluene). In either case, the vial was then placed at 80 °C oven for 24 h, the longer reaction time for the evaporation of toluene (solvent).

## 3. Results

**Figure 3:**
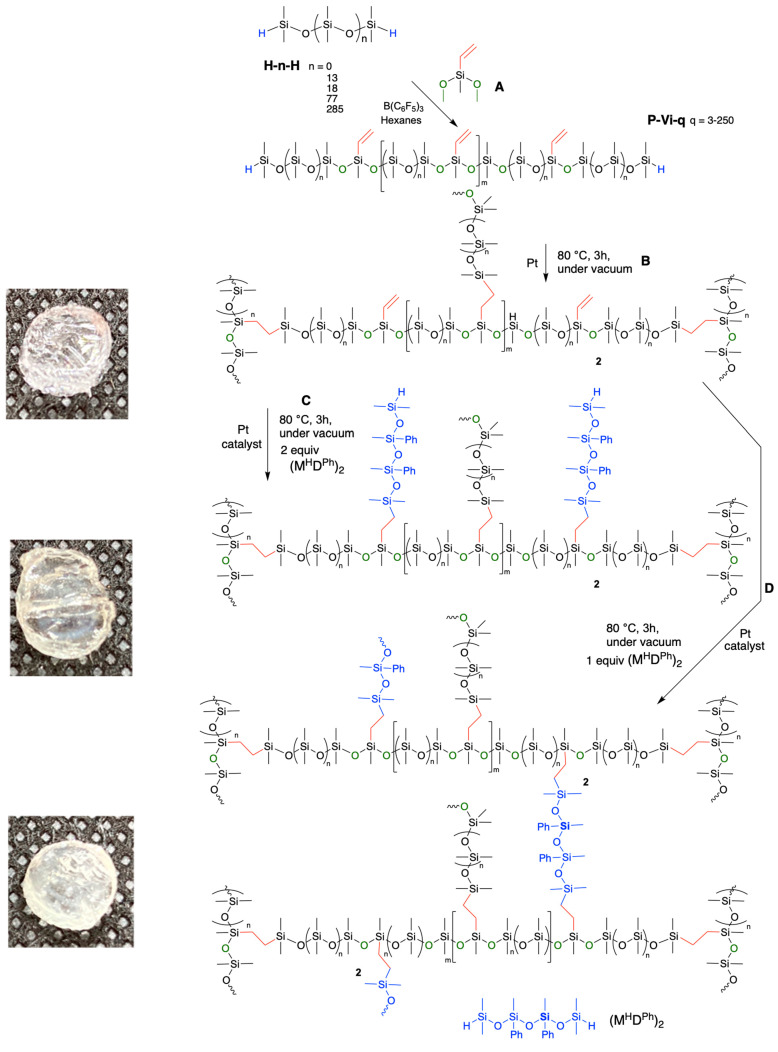
Synthesis of well-defined branched silicones using (**A**) Spatially controlled vinylsilicones using the Piers–Rubinsztajn reaction, shown for SiH terminated. (**B**) Self-curing of **P-Vi-n**. (**C**) Grafting of SiH branches using (M^H^D^Ph^)_2_. (**D**) Crosslinking using (M^H^D^Ph^)_2_.

Spatial control of vinyl groups on a silicone backbone was provided by the PR reaction between the difunctional vinyl monomer vinyldimethoxymethylsilane and αω-hydride-terminated PDMS of various chain lengths ranging from the disiloxane to longer chain linear polymers (MW 134 to 21,200 g mol^−1^ **H-D_n_-H** D = Me_2_SiO, **H** = M^H^ = Me_2_HSiO_2/2_; thus, **H-13-H** = M^H^D_13_M^H^) in the presence of the catalytic amount of B(C_6_F_5_)_3_ (0.02–0.20 mol% (Figure 3A) [28]. Using ^1^H NMR, the depletion of hydrosilane (SiH, ~4.7 ppm) or methoxy (SiOCH3, ~3.5 ppm) signals allowed one to demonstrate full consumption of reagents had occurred to give **P-Vi-q**, where q = spacing between adjacent vinyl groups. Thus, **P-Vi-3** = ((ViMeSiO)(Me_2_SiO)_2_)_m_).

**Figure 4:**
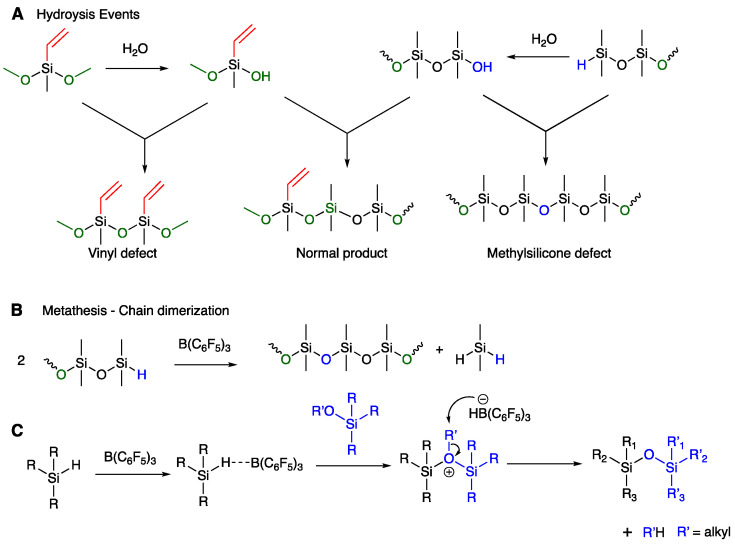
Origins of defects during chain extension. (**A**) Hydrolysis. (**B**) Metathesis. (**C**) Mechanism of the PR reaction.

The nature of the terminal functional groups was easily controlled simply by adjusting the [SiH]/[SiOMe] ratio (Table 1). Interestingly, at a stoichiometric [SiH]/[SiOMe] ratio, methoxy-terminated compounds were often obtained due to the occurrence of side reactions including metathesis that consumed some of the hydrosilanes (2 R_3_SiOSiMe_2_H→R_3_SiOSiMe_2_OSiR_3_ + Me_2_SiH_2_, Figure 4A,B) [29]. Three factors controlled the precision of the spatial location of vinyl groups: the *Đ_M_* of the telechelic silicone; loss of functional groups due to undesired metathesis; and the degree to which the reaction is maintained in a dry state [29]. Any defects are manifested in the differences, in some cases, between the expected vinyl frequency based on starting materials and the observed vinyl frequency in the product. For example, the expected vinyl% for the product of **H-13-H** should be about 7%, but in some cases was slower than expected due to chain extension from water (Figure 4A,B, entry 6 vs. 2, 10, Table 1) [29].

As with any AA + BB polymerization, the ultimate molecular weight was dependent on the precision with which the AA/BB stoichiometry was matched. One advantage with this chemistry is that, if water prematurely terminates the process, polymerization can be restarted using small quantities of a difunctional HSi compound (although, as noted above, this will lead to a defect in the vinyl spacing). Relatively high yields were obtained for these polymerizations (75–99%) except in the case of M^H^M^H^ which, due to its high volatility, was partly lost once exposed to the heat released during the exothermic Piers–Rubinsztajn reaction [30]. The vinyl concentrations (SiC*H* = C*H_2_*, ~5.8 ppm) in the resulting polymers varied from 0.4 to 29% in products with molecular weights ranging from 12,000 to 143,000 g mol^−1^ (^1^H NMR and GPC data refer to Tables S1 and S2 in the Supporting Information, SI).

The prevailing methodology for crosslinking in silicone elastomer synthesis is hydrosilylation, which involves the addition reaction between hydrosilane (SiH) and unsaturated bonds, in the presence of platinum catalyst, typically Karstedt’s catalyst [31]. Normally, these systems are sold as two-part pre-elastomer kits in which one part contains vinylsilicone and catalyst, while in the other part, hydrosiloxanes are found that are often the crosslinking partner. Polymers containing both functionalities are exceptionally rare, as neither traditional acid nor base catalyst technologies are compatible with both vinyl and HSi functionalities. The compounds produced fall in a convenient MW range of 10,000–60,000, within the 20,000–30,000 g mol^−1^ range that has been found to be convenient for many applications, once cured into elastomers [32]. These H/vinyl silicone polymers were able to self-cure to give bubble-free elastomers by addition of Karstedt’s catalyst (entries 1–4 Table 2, Figure 3B, ^1^H NMR in Table S3, SI). The Shore OO hardnesses of elastomers created from this homologous series ranged from 46 to 73 depending on overall MW and the spacing between vinyl groups.

**Figure 5:**
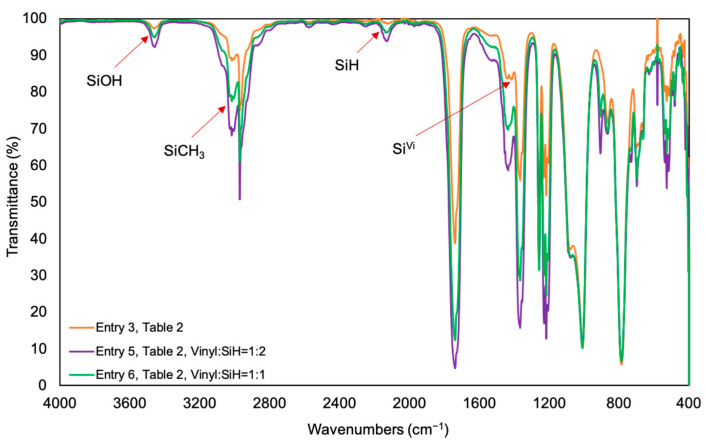
IR spectra for PMS-H03 modified elastomers using **P-Vi-28** self-crosslinked elastomers (Table 2). The orange curve shows the IR spectra of precursor elastomer (Entry 3, Table 2), the purple curve shows the IR spectra of PMS-H03 (Vinyl:SiH = 1:2) modified elastomer (Entry 5, Table 2) and the green curve shows the IR spectra of PMS-H03 (Vinyl:SiH = 1:1) modified elastomer (Entry 6, Table 2).

The elastomers prepared by self-hydrosilylation reactions possessed residual double bonds. These could be further modified to create new networks by swelling using different concentrations of the small telechelic hydrosilicone (HMe_2_SiOSiPhMe)_2_O (M^H^D^Ph^)_2_ into the elastomers and then again curing with hydrosilylation (Figure 3C,D). Use of 2 equiv (based on SiH) led to SiH functional elastomers and a small increase in hardness; use of 1 equiv led to harder elastomers (entries 5–8, Table 2). Note: the materials were more highly crosslinked and much harder than the precursor elastomer but shattered during Shore A measurements. When an excess of SiH was used (2 equiv), all vinyl groups were consumed, while with the use of 1 equiv of SiH/1 equiv of vinyl, not all vinyl groups reacted, which is expected because of the lower degrees of freedom of mobility in a network (^1^H NMR in Table S3, IR spectra in Figure 5). SiH groups were present in both products, suggesting that the limited degrees of freedom available to the short chains prohibits complete reaction.

The ultimate aim of this study was the creation of linear silicones with a spatially controlled distribution of high-density 3D branches. We hypothesized that spatial control would avoid clusters of functional groups, permitting full functionalization of the backbone. Spatially controlled vinyl-pendent linear silicone **P-Vi-14** with a molecular weight of 37,400 g mol^−1^, vinyl conc. of ~7%, and spacing chain length of 1100 g mol^−1^, DP~34 (that is, the macromonomer species is ~[(Me_2_SiO)_15_(MeSiVi)]_n_) served as the starting material. Dense branches were created using a series of iterative processes, alternating between hydrosilylation and then PR with a variety of monomers bearing different functionalities including SiH, SiOEt, and vinyl groups. Initially these reactions could be run neat. However, as the molecular weight increased, particularly above the entanglement limit of about 29,000 g mol^−1^ (reported entanglement limits for PDMS range from about 15,000–35,000 g mol^−1^; here, we use data from the seminal study of Mrozek et al. [25,33]) and branch density increased, it was necessary to dilute the system with hexanes [34]. Note that each iteration of PR then hydrosilylation could be undertaken in one pot, as the catalysts do not interfere with each other [35].

**Figure 6:**
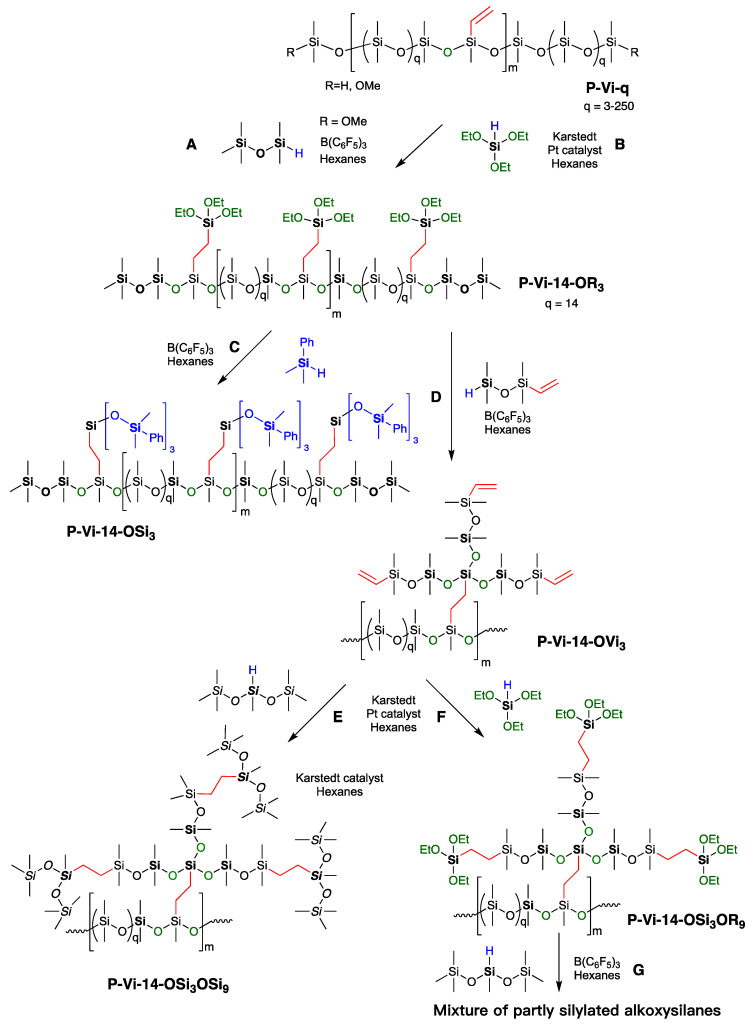
Synthesis of well-defined branched silicones by (**A**) capping residual SiOMe groups. (**B**) Hydrosilylation to introduce new SiOEt groups. (**C**) PR reaction to give branched siloxanes. (**D**) Chain extension to give trivinyl branches. (**E**) Dense silicone branches from hydrosilylation. (**F**) Dense alkoxysilane from hydrosilylation. (**G**) Failed attempt to extend the branches by hydrosilylation.

Selected examples of dendron-like branch growth are shown in Figure 6. The terminal methoxy groups on compound **P-Vi-14** were first capped using an excess of MM^H^ in the presence of B(C_6_F_5_)_3_ catalyst; this process can be performed during workup of compound **P-Vi-14** and avoids adventitious PR side reactions. Hydrosilylation with triethoxysilane in the presence of Karstedt’s catalyst creates trifunctional branches in **P-Vi-14-OR_3_** (Figure 6B,C). Note that, if desired, lower degrees of functionalization should be accessible by hydrosilylation with MeHSi(OEt)_2_ or Me_2_HSiOEt. ^1^H NMR spectra showed the complete consumption of the vinyl group (SiC*H = CH*_2_, ~5.8 ppm) and a newly emerged ethoxy peak (SiOC*H*_2_CH_3_, ~3.8 ppm). The alkoxy groups undergo ready silylation in a PR process, for example, with Me_2_PhSiH to give highly branched, phenyl-rich compound **P-Vi-14-OSi_3_**.

The iterative PR/hydrosilylation process can be repeated with compound **P-Vi-14-OR_3_**, for example, using vinyltetramethyldisiloxane to give alkoxysilane-free compound **P-Vi-14-OVi_3_**. The net effect is to convert single pendent vinyl groups in compound **P-Vi-14** into trivinyl-functional branches in compound **P-Vi-14-OVi_3_**. These vinyl groups, in turn, were converted to more highly branched silicones **P-Vi-14-OSi_3_OSi_9_**, or new alkoxysilanes **P-Vi-14-OSi_3_OR_9_**. It was not possible, in our hands, to obtain clean products from **P-Vi-14-OSi_3_OSi_9_** or a further PR reaction product **P-Vi-14-OSi_3_OR_9_**, which we ascribe to steric issues; a maximum efficiency of ~53% substitution was observed at 50 °C for 24 h in an excess of MD^H^M.

Some caveats must be attached to the synthetic sequence provided above. One must be exceptionally careful to store any of the alkoxysilane products completely dry. For example, although the conversion of **P-Vi-14** → **P-Vi-14-OSi_3_** led to the expected increase in molar mass, the analogous conversion of **P-Vi-14** → **P-Vi-14-OVi_3_** gave polymers with about 5x the expected molar mass. This is a consequence of very small amounts of water that lead to hydrolysis/condensation. Note that this problem is exacerbated at higher generations (researchers with better hands than ours are able to successfully manage this challenge [17,18]). Compound **P-Vi-14-OVi_3_OR_9_** has a molar mass by GPC that is appropriate based on its initial starting material **P-Vi-14** but lower than its immediate precursor **P-Vi-14-OVi_3_**. The molecular weight did not decrease. Instead, a fraction of the material became a resin that was no longer carried though the column. Note that, in addition at high branch densities, GPC can underestimate molecular weight because the behavior of linear calibrating polymers is quite different from these highly branched polymers, which can take up a globular structure [25,36].

## 4. Discussion

The Piers–Rubinsztajn reaction, combined with hydrosilylation, has previously been shown to be a valuable strategy to create structured networks [35]. Here, we demonstrate that it similarly permits the preparation of highly functional, highly branched polymers and macro-crosslinkers. Use of Skov’s technology permits precise spacing of vinyl functional groups between D units along a silicone backbone 1/3 to 1/250 (Table 1). The secondary level of control—DP of the macromer [(MeViSiO)_1_(Me_2_Si)_n_]_DP_—was more difficult to manage; depending on the specific starting material ratios chosen, [SiOMe]/[SiH] DPs ranging from 2 to 188 were realized. As expected, the highest MW were achieved with a stoichiometric balance that reduced the degree of premature termination.

The ideal spacing of vinyl groups was not always observed in these reactions. We attribute this observation to defects introduced by hydrolysis/condensation and metathesis; hydrolysis [29] and metathesis [28,37] reactions are promoted by B(C_6_F_5_)_3_. In both cases, the first formed SiOH groups can undergo condensation to give chain extension. SiH groups in the presence of B(C_6_F_5_)_3_ can also undergo metathesis reactions leading to longer polymers and coproduction of Me_2_SiH_2_. Either of these processes will lead to a defect in the chain with either a double or missing vinyl group (Figure 4A,B). Both of the processes leading to defects are less efficient/less rapid than the PR reaction. Thus, an advantage of the use of 1/1 [SiOMe]/[SiH] stoichiometry is a lower likelihood of defects. Note that we currently do not have the analytical capacity to detect these defects.

These reactions exhibit one unusual feature: low *Đ*_M_ [13]. Normally, one would expect ideal condensation polymer should normally have a dispersity approaching 2. Instead, the lower *Đ*_M_ suggests the propagation has a kinetic/chain growth component. The mechanism for the PR reaction involves two nucleophilic substitutions. Either one could be the rate determining step of the overall process, similar to traditional chain processes rather than condensations (Figure 4C) [22,38,39,40]. Chojnowski et al. have shown that related B(C_6_F_5_)_3_-catalyzed reaction of cyclic hydrosiloxanes is kinetically controlled [41]. Regardless, this constitutes an advantage of the process.

Once vinyl groups were located in precise locations on a silicone chain, it was relatively straightforward to use iterative hydrosilylation/PR cycles to grow dense branches that terminated with functional groups or, as we showed here, capped to give branched silicones (Figure 6). As with dendrimers, at higher generations, defects set in [42] and care must really be taken with the high-density triethoxysilyl branches to convert them to hydrolytically stable analogues, either silyl or vinyl. However, even after one generation, high molecular weight, highly functional, precise highly polymers are readily available.

Structure matters to all chemists. There is a paucity of data on the behavior of branched silicones. Charlesby created branched silicone by irradiation to create backbone radicals [43]. The method may have produced some lightly branched materials, but the author also recognized that their materials likely included crosslinked or uncontrollably branched components. The creation of these highly branched materials using a combination of PR and hydrosilylation reaction opens opportunities to fine tune the properties of both oils and elastomers. As the library of branched and highly branched [25] silicones is expanded, it will be possible to better identify the beneficial properties of both oils and elastomers that arise from precisely located vs. random branches.

## 5. Conclusions

A facile synthetic route to spatially controlled vinyl-pendent linear silicones, with vinyl spacing varying from 1/3 to 1/250 monomer units, low dispersities, and molecular weights of the obtained polymers ranging from 12,100 to 102,400 g mol^−1^ was reported. The terminal groups (SiH or SiOMe) of the polymers could by chosen by tuning the stoichiometry of the hydride-terminated PDMS and dimethoxymethylvinylsilane starting materials. The polymers with SiH termini underwent direct crosslinking using hydrosilylation to produce relatively soft elastomers containing residual vinyl groups that could be used for secondary functionalization or crosslinking. The vinyl groups were also used as loci from which dendritic branches were grown, eventually to make highly branched functional oils. The library of polymers produced, with precisely spaced mono- or dendritic multi-functional pendent groups, follows the paradigm of greater synthetic control to give narrower ranges of properties, and will permit an assessment of structure property relationships to allow the design of better silicone polymer fluids and elastomers.

## Figures and Tables

**Table 1:** The synthesis of spatially controlled vinyl silicones with SiH or SiOMe as terminal functional groups.

#	H-D_n_-H ^a^	M_n_ (g mol^−1^) ^b^	[SiH]/[SiOMe]	M_n_ (g mol^−1^) ^c^	*Đ*_M_	DP ^d^	Product Name P-Vi-q	Vinyl Conc. (%)	Yield (%)	Terminal Groups
1	**H-0-H**	134	1	N/A ^e^	N/A ^e^	-	**P-Vi-3**	31.5	66	OMe
2	**H-13-H**	1100	1	85,800	1.65	78	**P-Vi-16**	6.1	86	OMe
3	**H-18-H**	1500	1	102,400	1.11	68	**P-Vi-26**	3.9	75	OMe
4	**H-77-H**	5800	1	87,000	1.20	15	**P-Vi-200**	0.5	84	OMe
5	**H-0-H**	134	1.2	25,200	1.68	188	**P-Vi-4**	28.8	54	SiH
6	**H-13-H**	1100	1.2	26,900	1.68	24	**P-Vi-29**	3.4	86	SiH
7	**H-18-H**	1500	1.2	65,700	1.39	44	**P-Vi-28**	3.6	84	SiH
8	**H-77-H**	5800	1.2	13,300	2.09	2	**P-Vi-111**	0.9	89	SiH
9	**H-0-H**	134	0.83	N/A ^e^	N/A ^e^	-	**P-Vi-3**	35.4	63	OMe
10	**H-13-H**	1100	0.83	37,400	1.85	34	**P-Vi-14**	6.9	84	OMe
11	**H-18-H**	1500	0.83	23,600	2.15	16	**P-Vi-17**	5.7	99	OMe
12	**H-77-H**	5800	0.83	23,400	1.18	41	**P-Vi-83**	1.2	99	OMe
13	**H-285-H**	21,200	0.83	143,200	1.02	7	**P-Vi-250**	0.4	99	OMe

^a^ D = Me_2_SiO, M^H^ = Me_2_HSiO_2/2_ Thus, **H-0-H** = M^H^M^H^, **H-18-H** = M^H^D_18_M^H^. ^b^ M_n_ of the starting material obtained by ^1^H NMR end group analysis. ^c^ M_n_ obtained of the product obtained by GPC. ^d^ macromonomer repeat units [(ViMeSiO)_1_(Me_2_SiO)_n_]_DP_. ^e^ MW was too low to be detected by GPC.

**Table 2:** The synthesis of silicone elastomers using hydride-terminated, well-defined vinyl silicones via hydrosilylation.

	Self-Crosslinked Starting Polymer ^a^	Elastomers SVinyl Conc. (%) ^a^	MW (g mol^−1^) ^a^	Product Shore OO
1	**P-Vi-4**	28.8	25,200	46 ± 1
2	**P-Vi-29**	3.4	26,900	73 ± 2
3	**P-Vi-28**	3.6	65,700	64 ± 2
4	**P-Vi-111**	0.9	13,300	52 ± 2
	**Grafted copolymer networks** **Starting elastomers**		**Equiv ^a^ M^H^** **versus Si^Vi^**	
#				
5		entry 3	2	78 ± 2 ^b^
6		entry 3	1	82 ± 1 ^b^

**^a^** Compounds from Table 1 entries 5–8. ^b^ These materials are at the upper level of the Shore OO scale, but they were too brittle (they are unfilled) for measurement by Shore A—they cracked during measurement.

## Data Availability

Data is contained within the article or Supplementary Material.

## Supplementary Materials

The following are available online at https://www.mdpi.com/2073-4360/13/6/859/s1, Table S1. The synthesis of well-defined vinyl silicones with SiH or SiOMe as terminal functional groups (Table 1)—^1^H NMR data; Table S2. The synthesis of well-defined vinyl silicones with SiH or SiOMe as terminal functional groups (Table 1)—GPC data; Table S3. The synthesis of silicone elastomers using hydride-terminated, well-defined vinyl silicones via hydrosilylation; ^1^H NMR data, Calculation of vinyl concentration based on ^1^H NMR.

## Sample Availability

Samples of the compounds are not available from the authors.
