# Supplementary material for: Spatially Controlled Highly Branched Vinylsilicones
Source: Polymers (Basel). 2021 Mar 11;13(6):859. doi: 10.3390/polym13060859 (PMC8000532; doi:10.3390/polym13060859)
Supplement: Supplementary file 1 [file polymers-13-00859-s001.pdf]

Spatially Controlled Hyperbranched Vinylsilicones

Mengchen Liao, Yang Chen, Robert Bui and Michael A. Brook *

Supporting information

Table S1. The synthesis of well-defined vinyl silicones with SiH or SiOMe as terminal functional groups (Table 1) - ^{1}H NMR data

Entry	^{1}H NMR ($CDCl_3$, 600 MHz, δ)
1	5.78-6.05 (m, 3H), 3.46-3.47 (SiOCH_3, m, 0.48H), 0.07-0.23 (m, 16.08H) ppm.
2	5.78-6.03 (m, 3H), 3.47-3.48 (SiOCH_3, m, 0.18H), 1.54 (s, 1.74H), 0.07-0.27 (m, 94.68H) ppm.
3	5.77-6.02 (m, 3H), 3.46-3.47 (SiOCH_3, m, 0.27H), 1.54 (s, 0.88H), 0.06-0.26 (m, 149.35H) ppm.
4	5.77-6.04 (m, 3H), 3.47-3.48 (SiOCH_3, m, 3.13H), 1.54 (s, 12.12H), 0.07-0.16 (m, 1237.10H) ppm.
5	5.78-6.03 (m, 3H), 4.70-4.76 (Si*H*, m, 0.01H), 1.54 (s, 0.55H), 0.07-0.24 (m, 17.84H) ppm.
6	5.78-6.03 (m, 3H), 4.70-4.71 (Si*H*, m, 0.50H), 1.55 (s, 0.98H), 0.04-0.45 (m, 173.20H) ppm.
7	5.78-6.03 (m, 3H), 4.70-4.74 (Si*H*, m, 0.11H), 1.54 (s, 1.49H), 0.07-0.45 (m, 164.66H) ppm.
8	5.77-6.03 (m, 3H), 4.70-4.71 (Si*H*, m, 0.03H), 1.54 (s, 23.61H), 0.05-0.17 (m, 632.56H) ppm.
9	5.78-6.05 (m, 3H), 3.46-3.50 (SiOCH_3, m, 0.95H), 0.08-0.23 (m, 13.95H) ppm.
10	5.77-6.02 (m, 3H, SiC*H*=CH_2), 3.47-3.48 (s, 0.10H, SiOCH_3), 1.52 (s, 0.62H), 0.05-0.13 (m, 83.55H, SiCH_3) ppm
11	5.78-6.06 (m, 3H), 3.47-3.48 (SiOCH_3, m, 0.67H), 1.56 (s, 1.12H), 0.07-0.27 (m, 102.74H) ppm.
12	5.78-6.05 (m, 3H), 3.47-3.48 (SiOCH_3, m, 0.81H), 0.04-0.16 (m, 517.29H) ppm.
13	5.78-6.03 (m, 3H), 3.47-3.48 (SiOCH_3, m, 0.78H), 1.55 (s, 8.03H), 0.07-0.17 (m, 1621.13H) ppm.

Table S2. The synthesis of well-defined vinyl silicones with SiH or SiOMe as terminal functional groups (Table 1.)- GPC data

Entry	M_n (g mol^{-1})	M_w (g mol^{-1})	$Đ_M$
1	N/A	N/A	N/A

2	85,800	141,700	1.65
3	102,400	113,100	1.11
4	87,000	104,800	1.20
5	25,200	42,400	1.68
6	26,900	45,000	1.68
7	65,700	91,300	1.39
8	13,300	27,800	2.09
9	N/A	N/A	N/A
10	37,400	69,300	1.85
11	23,600	50,600	2.15
12	23,400	27,600	1.18
13	143,200	146,200	1.02

Calculation of vinyl concentration based on ^{1}H NMR

Sample calculation for Entry 10 in **Table 1.**

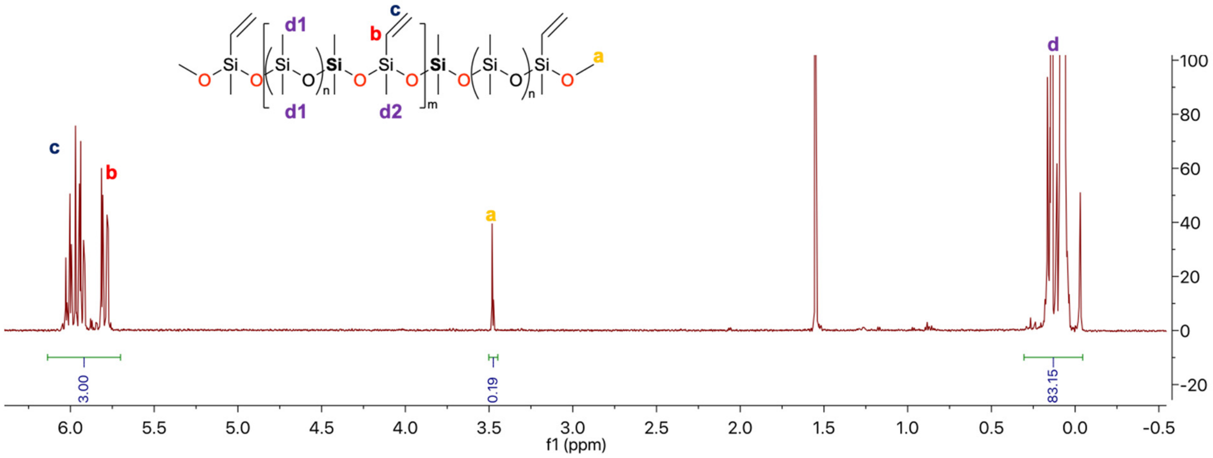

The vinyl integration was normalized to 3H. The fraction of vinyl monomer was calculated as:

$$\text{\# of d1 silicon for every repeating unit} = \frac{(\text{The integration of silioxane region-3})}{6} = \frac{(83.15-3)}{6} \approx 13$$

$$\text{Vinyl conc. (\%)} = \frac{1}{[\frac{\text{The integration of siloxane region-3}}{6}+1]} *100\ \% = \frac{1}{[\frac{83.15-3}{6}+1]} = 6.96\%$$

Table S3. The synthesis of silicone elastomers using hydride-terminated, well-defined vinyl silicones via hydrosilylation - ^{1}H NMR data (**Error! Reference source not found.**)

Entry[a]	^{1}H NMR ($CDCl_3$, 600 MHz, δ)
1	5.80-6.01 (m, 98H, CH_2=CH-Si), 0.83 (m, 4H, CH_2-CH_2), 0.08-0.15 (m, 606H) ppm.
2	5.78-5.94 (m, 0.8H, CH_2=CH-Si), 0.80-0.86 (m, 4H, CH_2-CH_2), 0.07 (s, 44H) ppm.
3	5.77-6.00 (m, 5H, CH_2=CH-Si), 0.79-0.88 (m, 4H, CH_2-CH_2), 0.07-0.08 (m, 193H) ppm.
5	7.55-7.57 (m, 10H, aromatic H), 7.32-7.43 (m, 18H, aromatic H), 4.74-4.76 (m, 4H, SiH), 0.81-0.88 (m, 4H, CH_2-CH_2), 0.31-0.32 (m, 19H, Si(Ph)CH_3), 0.15-0.17 (m, 36H, Si(Ph)OSi(CH_3)$_2$), 0.07-0.08 (m, 117H) ppm. (Vinyl groups remain when only 1 equiv of SiH was used due to side reactions such as metathesis and hydrolysis that consume SiH groups)
6	7.55-7.58 (m, 7H, aromatic H), 7.32-7.39 (m, 12H, aromatic H), 5.94-5.97 (m, 0.16H, CH_2=CH-Si), 4.74-4.76 (m, 2H, SiH), 0.80-0.89 (m, 4H, CH_2-CH_2), 0.31-0.32 (m, 13H, Si(Ph)CH_3), 0.15-0.17 (m, 23H, Si(Ph)OSi(CH_3)$_2$), 0.07-0.08 (m, 98H) ppm.
4	5.77-6.03 (m, 0.90H, CH_2=CH-Si), 0.80-0.89 (m, 4H, CH_2-CH_2), 0.07 (s, 123H) ppm.
